# Complete Genome Sequence of Bovine Pestivirus Strain PG-2, a Second Member of the Tentative Pestivirus Species Giraffe

**DOI:** 10.1128/genomeA.00376-14

**Published:** 2014-05-15

**Authors:** Paul Becher, Nicole Fischer, Adam Grundhoff, Hanspeter Stalder, Matthias Schweizer, Alexander Postel

**Affiliations:** a Institute of Virology, University of Veterinary Medicine, Hannover, Germany; b Institute for Medical Microbiology, Virology, and Hygiene, University Medical Center Hamburg-Eppendorf, Hamburg, Germany; c Heinrich Pette Institute, Leibniz Institute for Experimental Virology, Research Group Virus Genomics, Hamburg, Germany; d Institute of Veterinary Virology, Vetsuisse Faculty, University of Bern, Bern, Switzerland; e German Center for Infection Research (DZIF), Partner Site Hannover-Braunschweig, Hannover, Germany; f German Center for Infection Research (DZIF), Partner Site Hamburg-Lübeck-Borstel, Hamburg, Germany

## Abstract

We report the complete genome sequence of bovine pestivirus strain PG-2. The sequence data from this virus showed that PG-2 is closely related to the giraffe pestivirus strain H138. PG-2 and H138 belong to one pestivirus species that should be considered an approved member of the genus *Pestivirus.*

## GENOME ANNOUNCEMENT

The genus *Pestivirus* within the family *Flaviviridae* comprises the established species bovine viral diarrhea virus-1 (BVDV-1), BVDV-2, classical swine fever virus (CSFV), and border disease virus (BDV), as well as a growing number of tentative pestivirus species isolated from various domestic and wild ruminant species and from pigs (1, 2). Complete genomic sequences have been reported for strains of the approved pestivirus species and for four additional tentative species represented by a group of atypical pestiviruses (Hobi-like viruses) isolated from cattle and buffalo, a novel group of ovine and caprine pestiviruses (e.g., strain Aydin/04-TR), porcine Bungowannah virus, and the giraffe pestivirus H138 (2, 3). Based on comparative sequence analysis of N^pro^ and E2 coding sequences, the bovine pestivirus strain PG-2 is most closely related to the H138 virus that was isolated from a giraffe in Kenya in 1967 (4). PG-2 was obtained from a bovine cell culture transformed by *Theileria* spp. in the 1990s. The *Theileria* spp. and the original host cells (bovine T cells or macrophages) originated from Kenya (5).

Here, we report the complete genome sequence of pestivirus strain PG-2 obtained by next-generation sequencing. RNA was extracted from the supernatant of an infected cell culture using the ViralAmp RNA purification kit (Qiagen, Hilden, Germany). Illumina libraries were generated from 15 ng RNA using a modified protocol of the SCRIPT SEQ version 2 RNASeq kit (Epicentre Biotechnologies) as described previously (6). The diluted library (2 nM) was sequenced on an Illumina MiSeq instrument (2 × 250 bp paired-end run, 615,229 reads). *De novo* assembly and taxonomic classification of the assembled contigs were performed as recently described (6). Of the total reads, 54.4% were found to be of host origin. Nearly all (99.2%) of the remaining reads assembled into a single sequence contig encompassing the entire bovine pestivirus strain PG-2 genome (average coverage, 7,765×).

The genome of PG-2 comprises 12,264 nucleotides (nt) and contains one large open reading frame (ORF). The 5′ and 3′ noncoding regions (NCR) are 381 nt and 198 nt long, respectively. The ORF comprises 11,685 nt and encodes a polyprotein encompassing 3,894 amino acids. A comparison of full-genome sequences revealed that PG-2 is most closely related to the giraffe pestivirus strain H138 (82.3% identity), whereas the identities with representatives of the other approved and tentative pestivirus species were <70%. The genome of the noncytopathogenic strain PG-2 is 338 nt shorter than that of the cytopathogenic strain H138. This difference is mainly due to the presence of a cellular sequence (encoding part of the J-domain protein interacting with viral protein) in the NS2 coding region of the H138 genome (7), which is absent from the genome of PG-2.

The bovine pestivirus strain PG-2 represents the second member of the previously described tentative pestivirus species giraffe. The availability of the complete genome sequence of PG-2 will allow detailed phylogenetic analyses contributing to studies on pestivirus evolution and may provide useful information for pestivirus species demarcation and classification.

### Nucleotide sequence accession number.

The genomic sequence of pestivirus strain PG-2 has been submitted to GenBank (accession no. KJ660072).
